# A framework for the joint institutionalization of climate change mitigation and adaptation in city administrations

**DOI:** 10.1007/s11027-018-9789-9

**Published:** 2018-03-01

**Authors:** Christian Göpfert, Christine Wamsler, Werner Lang

**Affiliations:** 1 Institute of Energy Efficient and Sustainable Design and Building (ENPB), Technical University of Munich, Arcisstr. 21, 80333 Munich, Germany; 2 Lund University Centre for Sustainable Studies (LUCSUS), Lund University, Box 170, 22100 Lund, Sweden

**Keywords:** Climate policy integration, Institutionalization, Mainstreaming, Mitigation, Adaptation, Urban planning, Municipal planning

## Abstract

Cities are key actors in reducing both the causes of climate change (mitigation) and its impact (adaptation), and many have developed separate mitigation and adaptation strategies and measures. However, in order to maximize outcomes, both scholars and practitioners are increasingly calling for more integrated and synergetic approaches. Unfortunately, related research remains scarce and fragmented, and there is a lack of systematic investigation into the necessary institutional conditions and processes. Against this background, this paper develops a framework to assess and support the joint institutionalization of climate adaptation and mitigation—here called *adaptigation*—in city administrations. This pioneering framework draws upon four key features of bureaucracies: organizational structure, visions and goals, actors, and technology and tools. Illustrated by pilot applications to the cities of Würzburg (Germany) and Mwanza (Tanzania), the framework provides a robust basis for future research, policy recommendations, and the development of context-specific guidelines for national and local decision-makers and officials. It highlights the importance of (i) clearly defined procedures for the implementation of adaptigation into urban planning processes (e.g., with the active involvement of stakeholders in the form of working groups or roundtable discussions), (ii) locally relevant goals and visions, established in collaboration with stakeholders, and (iii) the creation of mitigation and adaptation structures that are supported by the appropriate level of human resources, both within and outside city administrations. In this context, global, supranational, and national institutions play an important role in supporting institutionalization by providing targeted funding and promoting adaptigation, which requires the development of integrated goals, visions, and legislation.

## Introduction

As climate change is a global challenge, the early scientific discourse has focused on the responsibilities and policy options of nation states (IPCC 2014). However, both research and practice show that municipalities are playing an increasingly crucial role (Kern et al. 2005; Bulkeley 2010; UN-Habitat 2010, 2011a; Romero-Lankao 2012; Bulkeley and Betsill 2013; Castán Broto and Bulkeley 2013; Wamsler et al. 2014). The reasons for this include intense local carbon emissions, cities’ social, economic, and technical vulnerabilities and, more generally, their legal obligations and opportunities to take effective action (Rosenzweig et al. 2010; Romero-Lankao 2012; Dietrich and Göpfert 2014; Reckien et al. 2014; Säwert 2016; Singer-Posern 2016).

For many years, or even decades, municipalities have been engaged in “mitigation” or “adaptation” activities. In Germany, for example, municipalities have implemented energy-saving measures to improve cost effectiveness, while urban development and planning authorities have sought to protect the urban microclimate through legally binding restrictions on new planning applications (Fickert and Fieseler 2002; Anguelovski and Carmin 2011). However, strategic approaches with a normative vision, concrete goals, and clear measures that are based on mitigation or adaptation concepts are the exceptions (Wamsler et al. 2014). Some recent first steps in this direction are seen in the development of municipal climate mitigation policies (Kern et al. 2005; Bulkeley et al. 2009; Anguelovski and Carmin 2011; Göpfert 2014; Reckien et al. 2014). Initial efforts were limited to the energy sector, with the later addition of other fields such as urban traffic or, very occasionally, some aspects of adaptation (Romero-Lankao 2012; Castán Broto and Bulkeley 2013; Göpfert 2014). The integration of climate adaptation concepts into municipal policy and administrative structures is even newer, but has increased in both scientific and municipal practice in recent years (Anguelovski and Carmin 2011; Deutsches Institut für Urbanistik and Universität Bielefeld 2013; Castán Broto and Bulkeley 2013; Knieling and Roßnagel 2014; Wamsler 2015a; Säwert 2016).

Today, climate change mitigation and adaptation are increasingly seen as two sides of the same coin, i.e., as complementary strategies. Both the scientific community and political institutions, from the international to the local scale, have started to discuss the challenges and the need to integrate these two policy fields (McKibbin and Wilcoxen 2003; Dang et al. 2003; Stehr and Storch 2005; Tol 2005; Wilbanks 2005; Klein et al. 2005, 2007; Fleischhauer and Bornefeld 2006; Ritter 2007; Biesbroek et al. 2009; Laukkonen et al. 2009; Martens et al. 2009; Locatelli 2010; Schüle and Lucas 2011; UN-Habitat 2011a, b; Dymén and Langlais 2013). The exploration of synergies between municipal policy, strategy, and measures by addressing mitigation and adaptation together is a subject of growing scientific and practical importance (Climate Alliance 2007; Klein et al. 2007; Goklany 2007; Mahammadzadeh and Biebeler 2009; Laukkonen et al. 2009; Schüle and Lucas 2011; Schüle et al. 2011, 2016; Deutscher Städtetag 2012; Moser 2012; Dymén and Langlais 2013; Dietrich and Göpfert 2014; Deutsches Institut für Urbanistik 2015a; Dietrich and Schiffmann 2015; Göpfert 2015; Landauer et al. 2015; Säwert 2016; Zentrum Stadtnatur und Klimaanpassung 2017).

Nevertheless, there is still a lack of systematic, integrative approaches to, and analyses of the joint institutionalization of mitigation and adaptation in local governments. Institutionalization, in this context, is defined as a process that is designed to instill mitigation and adaptation as persistent and consistent aspects of organizational culture, for example, by establishing a vision, goals, roles, rule-based standard operating procedures, and organizational routines, in order to strengthen the legitimacy, foster the stability, enhance the predictability, and support the sustainable inclusion of mitigation and adaptation as an integral part of city administrations (Meyer and Rowan 1977; Scott 2003; van Waarden 2003; Anguelovski and Carmin 2011). However, most research and many guidelines continue to focus either on the implementation of municipal climate mitigation or on adaptation structures, plans, and strategies (Kern et al. 2005; Deutsches Institut für Urbanistik 2011; Göpfert 2014; UN-Habitat 2014; Reckien et al. 2014, 2015; Wamsler 2015b; Runhaar et al. 2017). Others simply focus on synergetic strategies and measures, and examine potentially complementary, conflicting or neutral interdependencies between mitigation and adaptation (Deutsches Institut für Urbanistik 2015a; Landauer et al. 2015). The holistic operationalization or joint institutionalization of these policy fields has received very little attention.

Against this background, the objective of this research was to create a consistent heuristic framework, based on robust theory and empirically specified variables, to assess and support the joint institutionalization of adaptation and mitigation—here called *adaptigation*—in city administrations. We call this the *Adaptigation Institutionalization Framework*. It was developed based on an in-depth literature review and interviews with experts. In addition, for our pilot application, we tested the framework on the administrations of the cities of Würzburg (Germany) and Mwanza (Tanzania). Since there is a general consensus in the literature that cities with 50,000 to 1 million inhabitants (so called medium-sized, secondary, intermediate, or intermediary cities) should be given greater attention in the current climate change mitigation and adaptation discourse (UN-Habitat 2011a; Reckien et al. 2014, 2015; UCLG 2016; United Nations 2016; Nel et al. 2016), the framework is predominantly intended for such administrations. While large and megacities have often been regarded as pioneers in the institutionalization of climate mitigation and adaptation policies (Anguelovski and Carmin 2011), most of the world’s cities (i.e., medium-sized cities) have been neglected and lack advice and an understanding of the concepts.

## Methodology

**Fig. 1:**
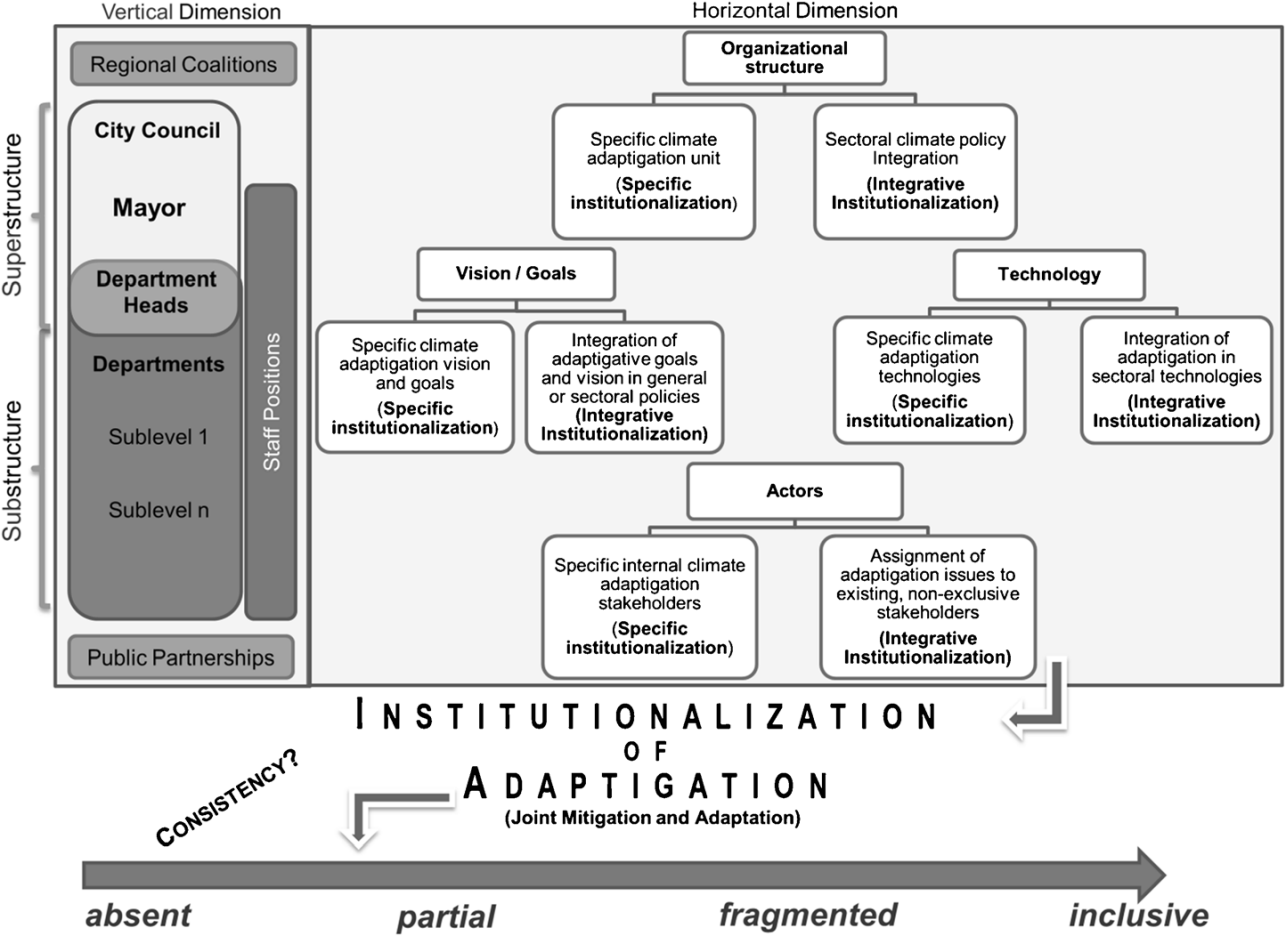
The *Adaptigation Institutionalization Framework* is an instrument for assessing the joint institutionalization of climate adaptation and mitigation (*adaptigation*) and its hierarchical and sectoral position in the city administration, based on four organizational features (structure, visions/goals, actors, and technology)

We used a three-step approach to construct the *Adaptigation Institutionalization Framework* (Kelle and Kluge 2010; Kuckartz 2014). First, some broad theoretical concepts were used as a heuristic to deduce a basic analytic coding scheme that formed the basis for a more detailed search of the literature (Section 3.1.1). Second, the assessment of current empirical and theoretical concepts and models on climate change provided further input (Section 3.1.2 and Fig. 1). Third, empirical data from existing studies and guidelines and our own field research helped to refine and revise the framework (Sections 3.2 and 3.3). The following sections (Sections 2.1 and 2.2) describe these steps in more detail.

### Theoretical foundations

The theoretical basis for the framework was deduced from established theories of sociological institutionalism, organizational culture, and organizational process models. First, we drew upon a key aspect of institutionalization process theory (North 1991; Czada 1995; Scharpf 2000), which argues that mitigation and adaptation must be implemented as an integral part of organizational culture (Martin and Meyerson 1988; Scott 2003; van Waarden 2003). When deeply internalized, they automatically become part of the organizational structure and decision-making processes (Selznick 1957; Scott 2003; Wolf 2005; Thoenig 2011). The second element concerns the four main features of bureaucracies used in the analysis, specifically organizational structure, goals and visions, actors, and technology (see Fig. 1). These factors are derived from *Leavitt’s diamond* (Leavitt 1965; Scott 2003) and the work of Malinowski (Gukenbiehl 2002). Third, in order to understand the decision-making processes and operating procedures used by municipalities, we draw upon an organizational process model, and the logic of appropriateness (Allison 1969; March 1999; Scott 2003; van Waarden 2003; March and Olsen 2008).

These general theoretical considerations were linked to climate governance via empirical frameworks regarding the concepts of climate policy integration and mainstreaming (Ahmad 2009; Beck et al. 2009; Mickwitz et al. 2009; Rietig 2012; Wamsler et al. 2014; Wamsler 2015a, b). This led to the creation of criteria designed to identify the horizontal and vertical institutionalization of mitigation and adaptation in different departments of an administration. We express the deep interconnections and inseparability of mitigation and adaptation by the term *adaptigation*, which is used to assess the joint implementation of mitigation and adaptation (Fig. 1), as introduced by Langlais (2009) and discussed by Dymen and Langlais (2013).

### Empirical data and analysis

In order to operationalize and adapt the analytic framework to the context of municipal administrative structures and processes, we first assessed the empirical literature (official studies and guidelines) on mitigation, adaptation, or both (Kern et al. 2005; Fleischhauer and Bornefeld 2006; BMVBS and BBSR 2009; Schüle et al. 2011, 2016; Schüle and Lucas 2011; Deutscher Städtetag 2012; Deutsches Institut für Urbanistik and Universität Bielefeld 2013; Göpfert 2014; Reckien et al. 2014; Wamsler 2015b, 2017; Deutsches Institut für Urbanistik 2015b; Hughes 2017). Second, we reviewed interviews with eight municipal officials responsible for climate-related issues^1^ to verify the practical relevance of the framework (Zentrum Stadtnatur und Klimaanpassung 2017). Empirical data were evaluated using a qualitative content analysis (Gläser and Laudel 2010; Kuckartz 2014). This led to the identification of eight variables, which were linked to the four features identified in the theoretical analysis, and confirmed their relevance.

Finally, a pilot test of the framework was run on the data collected in two case studies: Würzburg (Germany) and Mwanza (Tanzania). Based on an applied research approach (Greenwood and Levin 2006; Burns 2007), the main author participated in the mitigation and adaptation work of both cities for 7 years. For Würzburg, this involved active participation in all relevant meetings and decision processes, interviews, and document analyses. In Mwanza, most data were collected during interviews with municipal officials and active participation in meetings.

## Results

This section presents the *Adaptigation Institutionalization Framework*, which is an instrument designed to assess and support the joint institutionalization of climate mitigation and adaptation. The initial, basic framework linked the institutionalization assessment criteria to four main organizational features (organizational structure, goals and visions, actors, and technology/tools) (Section 3.1). Second, the examination of the empirical literature resulted in the operationalization of these features (Sections 3.2 and 3.3). Third, the applicability and the practical fit of the developed variables were tested in two case studies (Section 3.4). The resulting framework is presented in Fig. 1, and its different features/variables are described in the following sections. Table 1 presents a summary and brief explanation of the variables and their respective attributes.

**Table 1:** The *Adaptigation Institutionalization Framework*

Element of the framework	Description
Organizational features	*Organizational structure*: the formal organizational structure (*ORG*). *Goals/Visions:* quantitative goals (*GOA*), qualitative vision (*VIS*). *Actors:* internal individual actors (*IIA*), internal collective actors (*ICA*), external-internal collective actors (*ECA*). *Technology:* informal planning instruments (*IPI*), formal planning instruments (*FPI*).
Adaptigation assessment (joint institutionalization)	*Inclusive:* Both mitigation and adaptation are jointly implemented within the context of an organizational feature, for example, in the same organizational unit, via joint goals and visions, or allocating staff to both topics. *Fragmented:* Both mitigation and adaptation are implemented within the context of an organizational feature, but, for example, in different organizational units, with different goals and visions, in different internal committees or in the context of different standard operating procedures. *Partial:* Either mitigation or adaptation is implemented within the context of an organizational feature. *Absent:* Some issues may be addressed (e.g., urban greening or energy efficiency), but the topics of mitigation and adaptation are not explicitly implemented within the context of an organizational feature.
Horizontal institutionalization	*Specific:* Mitigation and/or adaptation is exclusively implemented within the context of an organizational feature, for example, in the form of a designated organizational unit, by creating a permanent post, or implementing a standard operating procedure that is designed to integrate mitigation and/or adaptation into urban planning processes. *Integrative:* Mitigation and/or adaptation is mainstreamed and embedded into existing organizational features, for example, in a non-climate specific unit (e.g., the urban planning department) as a secondary task, or in a sectoral standard operating procedure (e.g., binding planning regulations) that is the responsibility of a sectoral organizational unit.
Vertical institutionalization	*Superstructure:* Within the context of an organizational feature, mitigation and/or adaptation is hierarchically located at the level of the political or executive board. *Substructure:* Within the context of an organizational feature, mitigation and/or adaptation is hierarchically located at the department level (including its substructures).

### Theoretical background

#### Basic analytical features

The framework is based on four features of bureaucracies, which are generally used to systematically assess institutionalization processes (Fig. 1): organizational structure, visions and goals, actors, and technology. They describe that municipal administrations act within a framework structured by rules (organizational structure); they follow institutionalized patterns (visions and goals), where rules are matched with roles (actors). They implement standard operating procedures and routines (technology) consistent with the logic of appropriateness and the organizational process model (Allison 1969; March 1999; Scott 2003), rather than choosing a single best solution in every case. These four features were deduced from *Leavitt’s diamond* (Leavitt 1965; Scott 2003) and from general considerations regarding the systemic organization of administrations (Czada 1995; Gukenbiehl 2002; March and Olsen 2008; Pippke 2014).

- *Organizational structure*: This feature refers to the implementation of policies such as mitigation and adaptation within the formal structure (organizational units). It represents the “patterned or regularized aspects of the relationships existing among participants in an organization” (Scott 2003) that are used to implement the organization’s visions and goals. The reliability, stability, and effectiveness of a city administration are enhanced by persistent, formal, rule-based structures comprising defined roles, positions (mostly independent of specific individuals), routines, and hierarchies (Paulic 2014). Assuming a prevailing logic of appropriateness, it is vitally important to examine rules and structures to understand institutionalization processes, given that the institutional setting provides the frame for action (March 1999).
- *Visions and goals*: This feature comprises normative institutions and the value-driven implementation of policies through setting goals and creating a vision. The development of a qualitative vision is a vital element in institutionalizing climate-related issues (Gukenbiehl 2002). It is often influenced by the overall aims of institutions and organizations in a multi-level governance system (Thoenig 2011). This vision, which is likely to result in operationalized goals, could also be seen as a resource that policymakers can use in negotiations with other departments and stakeholders to enforce their preferences and implement concrete measures. Goals are defined as “conceptions of desired ends - ends that participants attempt to achieve through their performance of task activities” (Scott 2003). They become part of the administration’s organizational culture (Martin and Meyerson 1988). Following March (1999), appropriate and ambitious goals and visions are important to ensure that the organization performs well.
- *Actors*: This refers to the individual or collective actors making up the administrative body, who work to achieve its goals. The existence of specific, committed actors, and their role and power within the organization are seen as crucial factors for institutionalization processes (Scott 2003).
- *Technology*: This represents mechanisms “for transforming inputs into outputs” (Scott 2003) and includes procedures and tools designed to accomplish legally-required, or self-imposed tasks. The *Adaptigation Institutionalization Framework* incorporates decision-making and processing by the application of rules, and matching problems with standard operating procedures. Hence, technology is closely associated with defined procedures, roles, and rules that are applied in specific situations.

#### Horizontal, vertical, and joint dimensions

The review of insights from climate policy integration (Mickwitz et al. 2009; Rietig 2012) and mainstreaming approaches (Wamsler et al. 2014; Wamsler 2015a, b; Wamsler and Pauleit 2016) led to the addition of three analytic dimensions to each of the four features:

- The level of adaptigation (joint institutionalization of adaptation and mitigation). According to Langlais (2009), adaptigation “is a response to climate change that integrates a focus on adaptation with a focus on mitigation, to avoid conflicts and create synergies”.
- The location of mitigation and adaptation within different administrational units and their exclusive, adaptigation-specific implementation (horizontal institutionalization).
- Their location within the hierarchical structure (vertical institutionalization).

### Variables

The analysis of the empirical literature led to the identification of eight variables, which are key to operationalizing the four organizational features described in Section 3.1.1 (Fig. 1). These variables were selected based on their ability to address both mitigation and adaptation, and the literature that supports the choice of each variable relates to both fields.

#### Organizational structure

The formal implementation of mitigation and adaptation in the organizational structure, with clearly defined responsibilities, is crucial for institutionalizing climate-related issues in the long term (Kern et al. 2005; Deutsches Institut für Urbanistik 2011, 2015b, Schüle et al. 2011, 2016; Singer-Posern 2016). The organizational implementation of adaptation can also be connected to existing mitigation structures (Reckien et al. 2014; Deutsches Institut für Urbanistik 2015b).

The first variable, *ORG* (*organizational structure*), addresses the formal implementation of mitigation/adaptation in the organization. The focus is on the organizational units that are officially responsible for mitigation and adaptation respectively. Both the document analysis and the interviews found a lack of consistency regarding the implementation of climate issues in the organizational structure (cf. Deutsches Institut für Urbanistik 2011).

The review of the empirical literature revealed a wide spectrum of organizational localization. Mitigation is predominantly integrated into environmental departments (Kern et al. 2005; Schüle et al. 2011), while adaptation is more likely to be integrated into urban planning and development departments (Deutsches Institut für Urbanistik 2015b; Schüle et al. 2016), with some exceptions (Wamsler and Pauleit 2016). Healthcare, civil engineering, urban green space planning, economic development, or even public welfare departments can also lead the implementation of mitigation and adaptation (Deutsches Institut für Urbanistik and Universität Bielefeld 2013; Deutsches Institut für Urbanistik 2015b). In addition to the integration of climatic considerations into these sectoral entities, specific units, staff positions, or central offices designated as responsible for mitigation or adaptation can enhance institutionalization, or at least initiate related activities until other structures and mechanisms are in place (Schüle et al. 2011; Deutscher Städtetag 2012; Deutsches Institut für Urbanistik 2015b).

#### Visions and goals

The development and official implementation of a vision and goals play a crucial role in institutionalizing climate-related topics in municipal administrations. The following variables were selected to specify this feature:

- *VIS*: The existence of a qualitative mitigation/adaptation vision
- *GOA*: The existence of quantitative mitigation/adaptation goals

Goals and a vision provide a long-term perspective and are an essential part of the institutionalization process (Deutsches Institut für Urbanistik 2011; Schüle and Lucas 2011; Schüle et al. 2011, 2016; Göpfert 2014; Singer-Posern 2016). They should either be included in specific resolutions passed by the city council, or integrated into official urban planning strategies (Schüle et al. 2011; Wamsler 2015b), as mitigation and adaptation are generally considered as vital and inextricable elements of integrated urban development (BMVBS and BBSR 2009).

Examples of normative, guiding visions are resilient spatial structures in the case of adaptation (BMVBS and BBSR 2009; Schüle and Lucas 2011), or a “CO_2_-neutral” city in the case of mitigation. Examples of quantitative adaptation targets are limitations on areas that can be developed, or the definition of a specific percentage of green areas (Schüle and Lucas 2011). In the case of mitigation, most cities have set CO_2_ reduction goals (Deutsches Institut für Urbanistik 2011; an overview of 200 European urban areas is found in Reckien et al. 2014). Regarding adaptigation, Singer-Posern (2016) emphasize the importance of developing a guiding vision that encompasses both mitigation and adaptation. For instance, the German KoBe project (Empowerment of Municipalities at the Local Level to Adapt to Climate Change), conducted by the Wuppertal Institute for Climate, Environment, and Energy highlights the opportunity to develop a joint vision; examples include the “development of resilient energy infrastructures, emission-free, and climate-sensitive city” or the “energy efficient management of adaptation” (Schüle et al. 2016).

The lack of mitigation and adaptation regulation means that it is vital for councils to formulate and commit to local goals and a vision (Deutsches Institut für Urbanistik 2011; Deutsches Institut für Urbanistik and Universität Bielefeld 2013; Schüle et al. 2016). Without a political mandate, measures are conducted “under cover” (Schüle et al. 2016; Singer-Posern 2016), by integrating mitigation or adaptation issues into other sectoral policies and measures.

#### Actors

The empirical analysis found that appropriate personnel, intra-organizational cooperation, and cooperation with relevant external stakeholders are key to building mitigation and adaptation capacity. Therefore, we chose three variables to assess this feature:

- *IIA:* Internal individual actors
- *ICA:* Internal collective actors
- *ECA:* Internal–external collective actors

Internal individual actors are defined as administrative personnel who coordinate and support the implementation of mitigation and adaptation (Wamsler 2015b; Singer-Posern 2016). Dedicated personnel (“individual champions”, Wamsler 2017) with clearly defined responsibilities play a fundamental role in success (Deutsches Institut für Urbanistik and Universität Bielefeld 2013; Göpfert 2014; Singer-Posern 2016; interviews). These people typically hold positions such as “climate officer” or “city planner,” who is second-in-charge when it comes to climate issues. The interviewees and most of the reviewed studies highlight the advantages that come with the appointment of a central contact point, in the form of a person who is responsible for mitigation and adaptation (e.g., Kern et al. 2005; Schüle and Lucas 2011; Deutsches Institut für Urbanistik 2015b; Singer-Posern 2016).

Internal collective actors are defined as intra-organizational networks, such as interdepartmental, cross-cutting management structures (working groups, see e.g., Deutsches Institut für Urbanistik 2015b). When responsibilities within a municipal administration are fragmented, these structures support the institutionalization of climate-related issues through organizational learning, as participants exchange knowledge and develop a deeper commitment to goals and visions (Kern et al. 2005; Schüle et al. 2011, 2016; Deutscher Städtetag 2012; Deutsches Institut für Urbanistik and Universität Bielefeld 2013; Wamsler 2015b, 2017; Singer-Posern 2016; Hughes 2017). Internal cooperation can also increase opportunities to identify and successfully implement synergetic measures (Deutsches Institut für Urbanistik and Universität Bielefeld 2013).

Internal–external collective actors are defined by informal and formal committees (e.g., advisory councils such as climate committees, Deutsches Institut für Urbanistik 2015b; Singer-Posern 2016), which consist of both internal and external stakeholders. By participating in such committees, individual and collective actors, such as representatives of non-governmental organizations (NGOs) or housing associations can directly influence and enhance the implementation of a vision, goals, and technology (Schüle and Lucas 2011; Schüle et al. 2016). The implementation of these co-production structures has proven vital for institutionalization processes (Kern et al. 2005; Anguelovski and Carmin 2011; Deutsches Institut für Urbanistik and Universität Bielefeld 2013; Wamsler 2015b, 2017; Schüle et al. 2016).

#### Technology

Here, the focus is on city planning processes and associated tools, which, as the empirical literature and interviews highlighted, are crucial to the successful implementation of both mitigation and adaptation (Kern et al. 2005; Deutsches Institut für Urbanistik 2011; Deutsches Institut für Urbanistik and Universität Bielefeld 2013; Singer-Posern 2016; Zentrum Stadtnatur und Klimaanpassung 2017). We chose two variables to assess this feature:

- *IPI*: Informal planning instruments
- *FPI*: Formal planning instruments

Informal planning instruments are defined as key strategy papers, including the overall municipal climate policy. They are often specifically formulated as climate mitigation or adaptation concepts, or as general planning or development strategies, with the integration of climatic aspects (Kern et al. 2005; Schüle et al. 2011, 2016; Deutsches Institut für Urbanistik and Universität Bielefeld 2013; Göpfert 2014; Wamsler 2015b, 2017; Singer-Posern 2016).

Formal planning instruments refer to the institutionalization of climatic issues through their clearly-defined integration into standard operating procedures, in the form of internal and legal processes, such as administrative actions, or binding planning regulations (Kern et al. 2005; Fleischhauer and Bornefeld 2006; BMVBS and BBSR 2009; Deutsches Institut für Urbanistik and Universität Bielefeld 2013; Schüle et al. 2016; Singer-Posern 2016; Wamsler 2017). All interviewees highlighted that this was a top priority because of the lack of legal provisions.

### Empirical specification of assessment criteria

#### Operationalization of the adaptigation assessment

The term *adaptigation* describes the extent to which adaptation and mitigation are institutionalized into the four features (organizational structure, goals and visions, actors, and technology) of a city administration.

On this basis, the review of the empirical literature and the insights from the interviews identified four potential configurations: *absent*, *partial*, *fragmented*, and *inclusive*. Each of these configurations can be applied to each organizational feature. Logical expressions were used to clarify the attributes and for subsequent use in statistical analyses.

In the following, *MA* is the level of joint institutionalization, *x* is the variable, *M* is mitigation, and *A* is adaptation (see also Table 1):

- *Absent*: Neither mitigation nor adaptation is formally implemented. Some aspects may be implemented, but the official jargon is not used:

1 $$ {\displaystyle \begin{array}{c}{\mathrm{MA}}_x=\mathrm{absent}\\ {}\mathrm{for}\;{M}_x=0\;\Delta\;{A}_x=0\end{array}} $$

- *Partial:* Either mitigation or adaptation is implemented:

2 $$ {\displaystyle \begin{array}{c}{\mathrm{MA}}_{\mathrm{x}}=\mathrm{partial}\\ {}\mathrm{for}\;\left({M}_x=1\;\Delta\;{A}_x=0\right)\oplus \left({M}_x=0\;\Delta\;{A}_x=1\right)\end{array}} $$

- *Fragmented:* Both mitigation and adaptation are implemented, but separately:

3 $$ {\displaystyle \begin{array}{c}{\mathrm{MA}}_x=\mathrm{fragmented}\\ {}\mathrm{for}\;{M}_x=1\;\Delta\;{A}_x=1\;\Delta \neg \left({M}_x={A}_x\right)\end{array}} $$

- *Inclusive:* Both mitigation and adaptation are implemented together:

4 $$ {\displaystyle \begin{array}{c}{\mathrm{MA}}_x= inclusive\\ {}\mathrm{for}\;{M}_x={A}_x\end{array}} $$

#### Horizontal institutionalization

Like climate policy integration (Ahmad 2009; Beck et al. 2009; Mickwitz et al. 2009; Rietig 2012) and the mainstreaming approach (Wamsler et al. 2014; Wamsler 2015a, b; Wamsler and Pauleit 2016), and supported by insights from the empirical analysis (Schüle et al. 2011; interviews), the *Adaptigation Institutionalization Framework* distinguishes two forms of horizontal institutionalization. Either issues are implemented specifically and exclusively, with the sole purpose of either mitigation or adaptation (e.g., an organizational structure with a climate headquarter, Kern et al. 2005; Deutscher Städtetag 2012), or they are organizationally decentralized and mainstreamed in sectoral policies with a different primary focus (e.g., urban development concepts that include aspects of adaptation). In the following, *H* refers to horizontal institutionalization.

- *Specific* institutionalization:

5 $$ {\displaystyle \begin{array}{c}{H}_x=\mathrm{specific}\\ {}\mathrm{for}\kern.4em {\mathrm{MA}}_x\\ {}=\mathrm{implemented}\kern0.34em \mathrm{exclusively}\kern0.34em \mathrm{and}\kern0.34em \mathrm{designated}\kern0.18em \mathrm{as}\kern0.18em \mathrm{mitigation},\mathrm{and}\kern0.34em \mathrm{adaptation}\end{array}} $$

- *Integrative* institutionalization:

6 $$ {\displaystyle \begin{array}{c}{H}_x=\operatorname{int}\mathrm{egrative}\\ {}\mathrm{for}\kern.4em {\mathrm{MA}}_x\\ {}=\mathrm{secondary}\kern0.34em \mathrm{to}\kern0.34em \mathrm{another}\kern0.34em \mathrm{focal}\kern0.34em \mathrm{issue}\end{array}} $$

- *Special* case:

7 $$ {\displaystyle \begin{array}{c}{H}_x=\mathrm{differentiated}\\ {}\mathrm{for}\kern.4em {\mathrm{MA}}_x\\ {}=\mathrm{fragmented};{M}_x\kern0.18em \mathrm{and}\kern0.18em {A}_x\kern0.18em \mathrm{are}\kern0.18em \mathrm{implemented}\kern0.34em \mathrm{horizontally},\mathrm{but}\kern0.18em \mathrm{differently}\end{array}} $$

#### Vertical institutionalization

The interviews and review of the empirical literature showed that the location of issues and organizational units within the hierarchy of a city administration reflects their level of power and support (Kern et al. 2005; Deutsches Institut für Urbanistik and Universität Bielefeld 2013; Schüle et al. 2016; Singer-Posern 2016; Hughes 2017). For example, the administration of German cities consists of a political board (city council), a semi-political executive board, and several departments. The executive board is represented by the mayor and department heads. Departments are divided into organizational sublevels (Paulic 2014). Staff positions are located at all hierarchical levels. The empirical analysis underlined this variety in the vertical distribution of mitigation and adaptation issues (Schüle et al. 2011, 2016).

Within the context of vertical institutionalization, the framework distinguishes between the attributes of the “superstructure” (political and semi-political boards—possibly including staff positions) and the “substructure” (departments with several sublevels and staff positions). In the following, *V* refers to vertical institutionalization.

- *Superstructural* institutionalization:

8 $$ {\displaystyle \begin{array}{c}{V}_x=\sup \mathrm{er}\\ {}\mathrm{for}\kern.4em {\mathrm{MA}}_x\\ {}=\mathrm{implemented}\kern0.18em \mathrm{at}\kern0.18em \mathrm{city}\kern0.34em \mathrm{council}\kern0.34em \mathrm{or}\kern0.34em \mathrm{executive}\kern0.34em \mathrm{board}\kern0.34em \mathrm{level}\end{array}} $$

- *Substructural* institutionalization:

9 $$ {\displaystyle \begin{array}{c}{V}_x=\mathrm{sub}\\ {}\mathrm{for}\kern.4em {\mathrm{MA}}_x\\ {}=\mathrm{implemented}\kern0.18em \mathrm{at}\kern0.18em \mathrm{department}\kern0.34em \mathrm{level}\end{array}} $$

- *Special* case:

10 $$ {\displaystyle \begin{array}{c}{V}_x=\mathrm{differentiated}\\ {}\mathrm{for}\kern.4em {\mathrm{MA}}_x\\ {}=\mathrm{fragmented};{M}_x\kern0.18em \mathrm{and}\kern0.18em {A}_x\kern0.18em \mathrm{are}\kern0.18em \mathrm{implemented}\kern0.34em \mathrm{vertically},\mathrm{but}\kern0.18em \mathrm{differently}\end{array}} $$

### Pilot application

Here, we briefly present two case studies of the cities of Würzburg (Germany) and Mwanza (Tanzania), in order to illustrate the practical application of the framework to the administration of intermediary cities. Würzburg was chosen because of its partnership with the Technical University of Munich; the city provided open access to its data, which were needed to fully test the framework. In addition, Würzburg’s vulnerability to the effects of climate change, especially heat stress, is widely acknowledged (Künstler 2009; Karg et al. 2012) and has already been the subject of various research projects (see Stadt Würzburg 2017). The city of Mwanza, in Tanzania, was chosen because of its participation as a pioneering city in the municipal climate partnership program run by the German Federal Ministry for Economic Cooperation and Development (see Service Agency Communities in One World 2017), and the main author’s involvement in this partnership over a period of 6 years.

An overview of the results is shown in Tables 2 and 3. In short, the analysis found that the city of Würzburg already has a high level of joint institutionalization of mitigation and adaptation within the organizational structure and regarding the involvement of relevant stakeholders. Although the only concrete political commitments concern mitigation goals and visions, notably the creation of a specific unit, this has led, in recent years, to the bottom-up implementation of adaptation into participatory and technological structures.

**Table 2:** Summary of the Würzburg case study. For an explanation of the assessment criteria, see Table 1

Organizational feature	Description	Assessment criteria		
		Adaptigation	Horizontal	Vertical
Formal structure (*ORG*)	The *Stabsstelle Klimaschutz* unit is jointly responsible for mitigation and adaptation.	Inclusive	Specific	Substructure
Quantitative goals (*GOA*)	50% CO_2_ reduction, no explicit adaptation goal.	Partial	Specific	Superstructure
Qualitative vision (*VIS*)	Mitigation vision given in the “Würzburg 2030” document; no explicit adaptation vision.	Partial	Specific	Superstructure
Internal individual actors (*IIA*)	A climate protection officer, responsible for both mitigation and adaptation.	Inclusive	Specific	Substructure
Internal collective actors (*ICA*)	Informal *Arbeitskreis Klima* committee, members include administrators and politicians.	Inclusive	Specific	Superstructure
Internal-external collective actors (*ECA*)	The advisory committee *Klimabeirat* with participants from the administration, politicians, universities, and NGOs.	Inclusive	Specific	Superstructure
Informal planning instruments (*IPI*)	Focal climatic concept: *Klimaschutzkonzept*.	Inclusive	Specific	Superstructure
Formal planning instruments (*FPI*)	Adaptation is part of the formal urban planning process (standard operating procedure), mitigation is already integrated.	Fragmented	Integrative	Superstructure

**Table 3:** Summary of the Mwanza case study. For an explanation of the assessment criteria, see Table 1

Organizational feature	Description	Assessment criteria		
		Adaptigation	Horizontal	Vertical
Formal structure (*ORG*)	Environmental units at different sublevels with explicit responsibility for mitigation and adaptation (from the municipal environment department to ward and *mtaa* level)	Inclusive	Integrative	Substructure
Quantitative goals (*GOA*)	Various qualitative objectives within the National Climate Change Strategy, but no explicit quantitative goals for Mwanza.	Absent	Absent	Absent
Qualitative visions (*VIS*)	Enhancing climate resilience as specified in the National Climate Change Strategy.	Inclusive	Integrative	Superstructure
Internal individual actors (*IIA*)	Environmental management officers at different sublevels.	Inclusive	Integrative	Substructure
Internal collective actors (*ICA*)	Environmental management committee with members from different wards, dealing with both mitigation and adaptation.	Inclusive	Integrative	Superstructure
External-internal collective actors (*ECA*)	Decentralized, community-based organizations in different wards that discuss mitigation and adaptation issues.	Inclusive	Integrative	Superstructure
Informal planning instruments (*IPI*)	The National Climate Change Strategy, supplemented by local by-laws.	Inclusive	Integrative	Superstructure
Formal planning instruments (*FPI*)	Climate issues are considered in the context of urban planning (standard operating procedures). The environmental department gives advice to the planning department.	Inclusive	Integrative	Superstructure

Unlike Germany, in Tanzania, environmental policies and organizational structures (from the ministerial to the local *mtaa*^2^ level) were found to be coordinated centrally at national level. Examples include the National Climate Change Strategy, or the monitoring and evaluation framework for climate change adaptation in Tanzania, which are mandatory for every city (The United Republic of Tanzania 2012a, b). Additionally, as the reduction of greenhouse gas emissions is not an obligation, adaptation has been declared the highest priority. Most national directives are implemented in local by-laws. Hence, assessing the institutionalization of adaptigation requires a deeper investigation of how national directives and obligations are implemented.

Overall, the main insights obtained from the application of the framework to the two case studies are that (i) horizontal institutionalization is predominantly specific in Würzburg and integrative in Mwanza and (ii) the level of adaptigation is predominantly inclusive in both cities. The issues of mitigation and adaptation are seen as holistic and deeply interconnected, regardless of the dominant type of horizontal institutionalization: in Würzburg issues are specifically allocated to structures, while in Mwanza issues are integrated within a broader, environmental context.

## Conclusion

The *Adaptigation Institutionalization Framework* is a heuristic, analytic model, developed both for assessing and supporting the joint institutionalization of mitigation and adaptation in municipal administrations, especially medium-sized cities. The framework can also be used by the leaders, decision-makers, and officials of megacities as a heuristic instrument to initiate discussions about how the institutionalization of climate policies is structured in their administrations. The operationalization of the concept of adaptigation provides an urgently needed, innovative approach for science, policy, and in practice.

### Scientific research within and between cities

The framework can be used as a theoretical and empirically grounded instrument for conducting single and comparative (qualitative and quantitative) research studies. At city level, the framework is shown to be a useful basis for investigating interrelations, consistencies, or ambiguities between organizational features and variables. For example, a lack of consistency would manifest in ambitious goals that lack the necessary personnel to reach them (cf. Romero-Lankao 2012). In addition, it provides a heuristic for creating typologies of, and comparisons between, cities based on their level of adaptigation, and to generate hypotheses, for example, regarding correlations or interdependencies between the institutionalization of adaptigation, and the factual outcomes of concrete measures.

### Practical application and strategy recommendations

The framework has shown to have practical application and is able to create heuristic insights for ongoing processes and developments within city administrations. In addition, it can guide targeted capacity development to support the institutionalization of adaptigation. In fact, one of the underlying paradigms of the framework is the focus on, and links to capacity building (cf. Jänicke et al. 2003; Göpfert 2014). All four organizational features can be viewed in relation to how they can enhance action capacities for climate mitigation and adaptation.

**Fig. 2:**
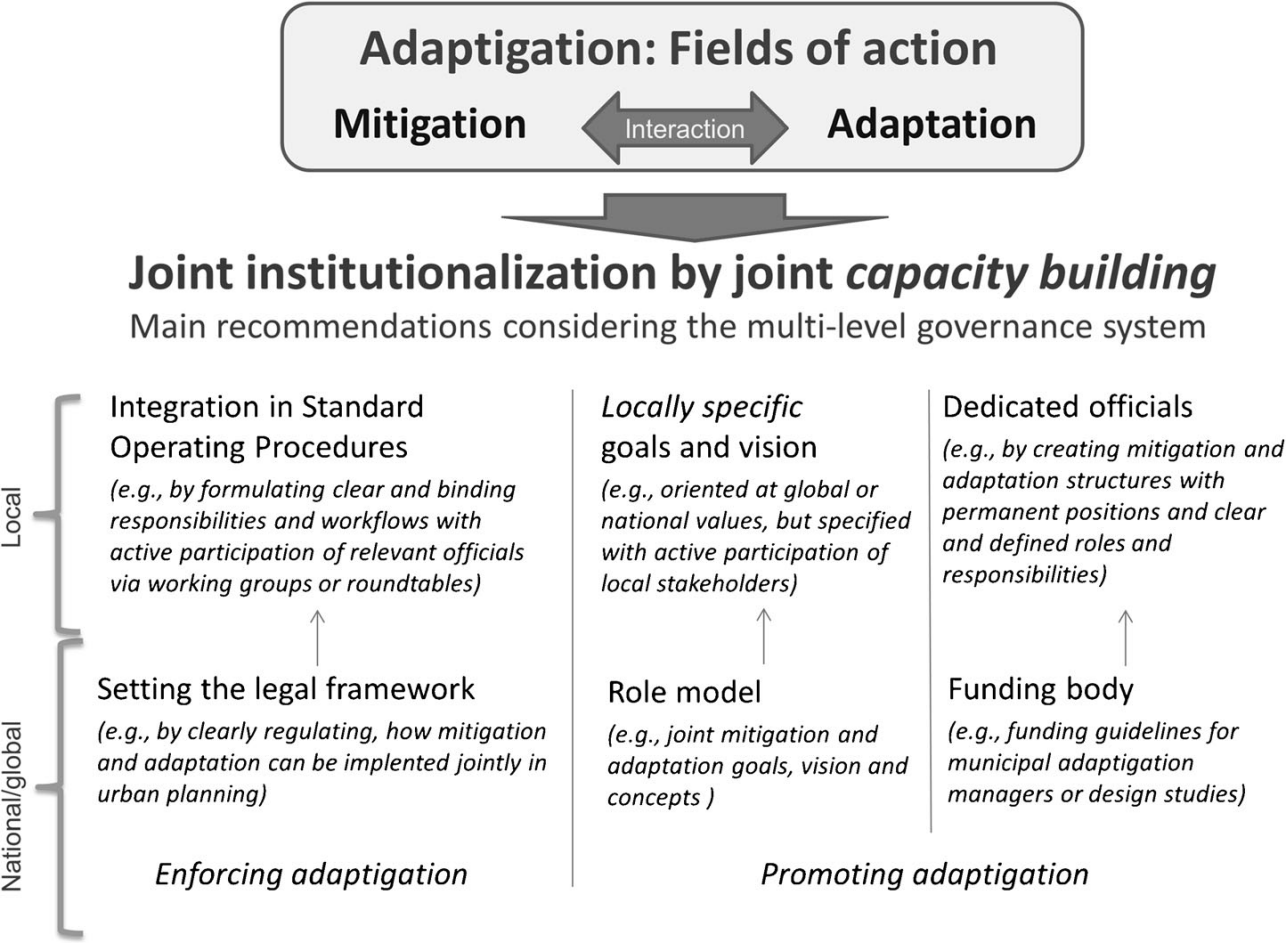
Recommendations to enhance the joint institutionalization of mitigation and adaptation in city administrations

The creation and execution of climate responses is likely to be more effective if mitigation and adaptation are jointly institutionalized in the organizational and political structures of city administrations. This means that decision-makers and politicians at all levels (from local to global) should not only consider potential interrelations and synergies between mitigation and adaptation measures, but also make the organizational setting more effective by thinking about the joint implementation of the two issues within the administration. In this context, our findings show that political and intra-organizational commitment to, and support for climate mitigation and adaptation are crucial for institutionalization. Given the very different paths that mitigation and adaptation processes have, as yet, taken in city administrations, it is clearly important to raise related awareness at both the administrative and political levels. Accordingly, the study resulted in three main strategy recommendations, addressing the identified key processes of joint institutionalization presented in Fig. 2:

- *Integration into standard operating procedures.* The ability of a city to fulfill its different tasks requires defined procedures, roles, and rules in specified situations. The importance of these factors, together with legitimate power (such as mandatory legal and internal directives), increases when there is a high level of uncertainty, for instance, with respect to technology (no clear directives and many interpretations of how to carry out tasks, etc.). In this case, the power of (certain) departments increases, and the power of political leaders decreases (cf. Scott 2003). In this context, the integration of adaptigation into the standard operating procedures of urban planning processes and associated action programs across different departments seem to be key for successful climate policy outcomes. Some important preconditions for this integration are (1) the definition of clear and binding organizational responsibilities and workflows, (2) co-production structures such as internal cross-sectoral working groups and/or roundtables, (3) interorganizational networks, such as climate committees for exchanging knowledge and fostering commitment, and (4) dedicated and well-trained personnel who hold a permanent position (possibly with defined adaptigation tasks) in the organization.
- *Locally specific goals and visions.* Generating a deep commitment to adaptigation within the administration, and from relevant external stakeholders is crucial and seems to depend, among other things, upon the ability to formulate joint local goals and develop a vision with active participation from those concerned (a bottom-up process). Establishing clear structures that allow for such knowledge co-production seems to be crucial for internalizing adaptigation within the administration. In addition, it can foster political consistency and reliability.
- *Dedicated officials.* The findings also suggest that officials at all hierarchical levels too often do not consider climatic issues because of a lack of awareness and knowledge regarding potential actions. This issue could be addressed by supporting dedicated staff who have the task of coordinating and promoting inter-departmental actions and information-sharing structures for adaptigation. Related organizational responsibilities and job specifications need to be clearly defined—either by creating a specific administrative unit, or by officially integrating the issues of mitigation and adaptation into existing, subject-related units and associated mandates. Within these units, the creation of permanent positions with clear and defined roles and responsibilities for adaptigation also seems to be crucial for successful institutionalization and long-term commitment (as opposed to temporary, partial engagement). Capacity building could reduce the need for additional staff/resources and reduce dependence on external experts (a form of human stockpiling).

These three processes can (and should) be supported by national, supranational, and global governance structures (Fig. 2). Higher-level governmental institutions can support the integration of adaptigation into standard operating procedures and enhance standardization, by clearly regulating how mitigation and adaptation can be implemented jointly. Furthermore, municipal policy is often oriented by national or global goals and visions; therefore, higher-level institutions are encouraged to explicitly promote the joint integration of mitigation and adaptation. Finally, funding should be provided at national and supranational levels (e.g., the European Union) to support the creation of adaptigative structures, such as adaptigation managers or design studies.

Although the framework is not designed to provide detailed, operational guidelines that specify context-specific actions, national and local decision-makers as well as officials can use it when considering how to improve the joint institutionalization of mitigation and adaptation strategies in city administrations. It is a robust starting point for obtaining an overview of the factors that should be considered when institutionalizing adaptigative structures. Even if it does not dictate, for instance, context-specific elements such as the horizontal implementation of administrative units (either as specific climate departments, or integrated into planning departments), it helps to focus on the key aspects that enhance the implementation of effective organizational structures and procedures. It highlights, for instance, that unclear or ill-defined goals should be made clear and acceptable (for instance, by developing an overall vision and specifying agreed goals); vague, temporary jobs should be turned into permanent positions with clear roles and responsibilities (e.g., the creation of a Climate Protection Officer position); and unclear technology should be transformed into standard operating procedures (e.g., by listing measures that fall under the scope of the climate mitigation/adaptation plan, or by integrating climate-related issues into formal planning procedures).

### Further research and applications

First and foremost, the framework developed in this paper is based on established theory and empirical data and therefore provides a robust basis for further research. Examples include in-depth case studies or quantitative surveys that can be used to develop context-specific guidelines and strategy recommendations for municipal decision-makers and officials. In addition, further applications to empirical data could provide useful insights for answering questions and identifying patterns regarding the pre-conditions and main requirements for generating and implementing synergetic measures, the best organizational design for particular settings, and limitations on the joint institutionalization of mitigation and adaptation.

We conclude that the *Adaptigation Institutionalization Framework* presented here provides a solid foundation for advancing current knowledge, and can be applied to a broad range of scientific and practical situations. Its relevance and international significance is based on its clear links with organizational theory, policy integration, and mainstreaming approaches in relation to the concepts of mitigation and adaptation.

## Footnotes

1 These interviews were conducted in 2017 by members of the Centre for Urban Ecology and Climate Adaptation at the Technical University of Munich.

2 The Swahili term *mtaa* can be translated as “neighborhood” or “street-level”.
